# Ethylene glycol poisoning in a free-roaming cat associated with suspected animal cruelty: a forensic veterinary case report in South Korea

**DOI:** 10.3389/fvets.2026.1846573

**Published:** 2026-05-15

**Authors:** Ah-Young Kim, Minji Kang, Hyeyoung Choi, Meejung Park, Ji-Su Baek, BokKyung Ku, Kyunghyun Lee

**Affiliations:** 1 Animal Disease Diagnostic Division, Animal and Plant Quarantine Agency, Gimcheon, Republic of Korea; 2 Toxico-Chemistry Division, Seoul Institute, National Forensic Service, Seoul, Republic of Korea

**Keywords:** animal cruelty, antifreeze poisoning, cat, ethylene glycol, forensic veterinary pathology, Korea, toxicology

## Abstract

Ethylene glycol (EG) is a common cause of fatal poisoning in companion animals and may be involved in intentional or negligent animal cruelty. We describe a forensic veterinary investigation of a free-roaming cat submitted by the Seoul Gangbuk Police in relation to an alleged violation of the Korean Animal Protection Act. Complete necropsy revealed focal gastric erosion and bilaterally congested kidneys, while histopathology of the kidneys showed acute tubular epithelial necrosis with abundant intraluminal calcium oxalate crystal deposition. Toxicological analysis of gastric tissue using a validated GC–MS method demonstrated the presence of ethylene glycol and its primary metabolite glycolic acid, confirming ethylene glycol exposure and, together with the gross and microscopic renal lesions, supporting antifreeze poisoning as the cause of death. PCR testing of pooled tissue samples was positive for feline parvovirus and feces were positive for feline coronavirus, but these infections were not considered the proximate cause of death. This case highlights the value of forensic veterinary pathology and toxicology in objectively determining cause of death in suspected animal cruelty investigations and demonstrates that targeted analysis of gastric tissue can provide definitive evidence of ethylene glycol exposure.

## Introduction

Animal cruelty investigations increasingly require collaboration between law enforcement authorities and veterinary forensic specialists to establish the cause and manner of death in companion animals (1–4). Veterinary forensic pathology and toxicology provide the scientific basis for interpreting injuries and deaths within a legal framework, including cases of suspected poisoning (1–3).

Ethylene glycol, the principal component of many automotive antifreeze products, is a well-recognized cause of acute intoxication in cats and dogs, leading to central nervous system depression, severe metabolic acidosis, acute kidney injury, and death (5–9). In cats, the minimum lethal dose of undiluted ethylene glycol has been reported to be approximately 1.4 mL/kg, and most untreated animals die within 24–72 h after ingestion (5–9). Although the clinical features and treatment of ethylene glycol poisoning in companion animals have been widely described, detailed forensic case reports that integrate necropsy, histopathology, and toxicological confirmation within the context of an official criminal investigation remain limited (5–7, 10).

Recent Korean studies have underscored the importance of toxicological analysis in fatalities with suspected companion animal cruelty, documenting a variety of toxicants including rodenticides, pesticides, and pharmaceuticals (11, 12). Here, we report a fatal case of ethylene glycol poisoning in a free-roaming cat in Seoul, South Korea, examined as part of a police investigation into alleged animal cruelty. We focus on the forensic aspects of case handling, including chain of custody, differential diagnosis, and interpretation of toxicological findings, to illustrate how veterinary expertise can support legal decision-making in suspected poisoning cases.

## Case history

On 4 December 2023, the Seoul Gangbuk Police Station received a report of dead cats in a public park area and opened an investigation under a suspected violation of the Korean Animal Protection Act. Two free-roaming cats were collected; one adult female free-roaming cat was selected for detailed forensic examination based on the available clinical information and carcass condition. According to the caretaker, this cat had been managed as a community cat and was admitted to a private veterinary hospital with acute onset ataxia, severe lethargy, and signs consistent with acute kidney injury; the cat died approximately 7 hours after admission despite supportive care. The police formally requested a postmortem examination from the Animal and Plant Quarantine Agency (APQA) to clarify the cause of death, and the carcass was transported under sealed conditions and registered as a forensic case.

## Materials and methods

### Necropsy and sampling

A complete forensic necropsy was performed according to recommended veterinary forensic protocols, with thorough documentation of external and internal findings and photographic recording of relevant lesions.

Signalment, body weight and body condition score (BCS) were recorded, and representative tissue samples were collected from brain, heart, lung, liver, spleen, kidneys, intestine and stomach.

For histopathology, tissues were fixed in 10% neutral buffered formalin, routinely processed, embedded in paraffin, sectioned at 3–5 μm and stained with hematoxylin and eosin (H&E).

For special staining, calcium deposits in the kidney were visualized using the Yasue silver impregnation method. Paraffin-embedded renal sections (4–5 μm) were deparaffinized in xylene, rehydrated through graded ethanol to distilled water, and incubated in 5% aqueous silver nitrate solution under strong visible light until black staining of calcium deposits became apparent. Sections were then rinsed thoroughly in distilled water, counterstained lightly with hematoxylin, dehydrated through graded ethanol, cleared in xylene, and mounted with a synthetic resin.

### Virological testing

To assess potential infectious causes of death and co-morbidities, PCR assays were conducted on pooled tissue samples (brain, spleen, lung, heart, kidneys, liver and intestine) and feces. Targets included feline immunodeficiency virus (FIV), feline herpesvirus (FHV), feline parvovirus (FPV), feline coronavirus (FCoV), feline leukemia virus (FeLV) and feline calicivirus (FCV), using in-house protocols routinely applied at APQA.

### Toxicological analysis

Toxicological examinations were performed at the National Forensic Service (NFS), Seoul Institute, Toxico-chemistry Division, using a validated gas chromatography–mass spectrometry (GC–MS) procedure adapted from Meyer et al. Ethylene glycol (analytical grade) was purchased from Junsei Chemical (Tokyo, Japan), while glycolic acid and N, O-bis(trimethylsilyl)trifluoroacetamide (BSTFA) with 1% trimethylchlorosilane (all analytical grade) were obtained from Sigma-Aldrich (St. Louis, MO, USA); all other reagents were also of analytical grade.

For toxicological examination, gastric tissue was selected as the analytical matrix because it was available for analysis and was likely to contain residual unmetabolized ethylene glycol shortly after ingestion. In the present case, the analytical approach was used for qualitative confirmation of ethylene glycol and glycolic acid in gastric tissue. Although authentic reference materials and a deuterated internal standard were available, a matrix-specific quantitative calibration curve for feline gastric tissue was not established for this case; therefore, concentrations of ethylene glycol and glycolic acid were not reported.

Gastric tissue (0.5 g) was placed in a microtube, mixed with 1 mL distilled water, and sonicated for 5 min. The homogenate was centrifuged at 12,000 rpm for 5 min, and 50 μL of the supernatant was transferred to a new tube and deproteinized with 500 μL acetonitrile. An internal standard solution of gamma-hydroxybutyrate-d6 (GHB-D6; 10 mg/L) was added at 50 μL, followed by centrifugation at 12,000 rpm for 5 min. The supernatant was transferred to a glass tube and evaporated to near dryness under a gentle stream of nitrogen, avoiding complete dryness. Derivatization was performed by adding 50 μL ethyl acetate and 30 μL BSTFA, followed by incubation at 90 °C for 15 min and cooling to room temperature before transfer to GC vials.

GC analysis was carried out on an Agilent 7890A gas chromatograph coupled to an Agilent 5975C MSD (Agilent Technologies, Santa Clara, CA, USA), equipped with an HP-5MS capillary column (30 m × 0.25 mm i.d., 0.25 μm film thickness). The injection port was maintained at 230 °C, the injection volume was 1 μL in splitless mode, and helium was used as the carrier gas at 1.0 mL/min. The oven temperature program was: initial 70 °C (1 min), ramped at 10 °C/min to 150 °C (1 min), then 30 °C/min to 290 °C (10 min). The mass spectrometer operated in electron-impact ionization and full-scan mode, with an ion source temperature of 280 °C. Trimethylsilyl (TMS) ether derivatives of ethylene glycol, glycolic acid, and GHB-D6 were monitored, and identification was based on retention time and characteristic fragment ions.

In addition to ethylene glycol and glycolic acid, the toxicological screening panel included cyanide, organophosphates, organochlorines, carbamates, anticoagulant and non-anticoagulant rodenticides, barbiturates, benzodiazepines, phenothiazines, salicylates and various alkaloids, in line with previously reported forensic protocols for suspected animal poisoning.

### Chain of custody and documentation

All specimen transfers, analyses and reporting steps were recorded in a case file that included the police request, laboratory accession number, bench notes and final diagnostic report, in accordance with published minimum standards for forensic investigations and national guidelines on forensic veterinary examinations.

## Results

### Necropsy findings

The cat was an adult neutered female with a body weight of 4.84 kg and a body condition score of 6/9. Externally, there was no evidence of trauma, hemorrhage, or restraint-related lesions. On internal examination, the stomach contained a small amount of mucoid material and exhibited focal mucosal erosion. Both kidneys showed generalized vascular congestion. Other organs, including the heart, lungs, liver, spleen, and brain, did not exhibit remarkable gross lesions.

### Histopathology

**Figure 1:**
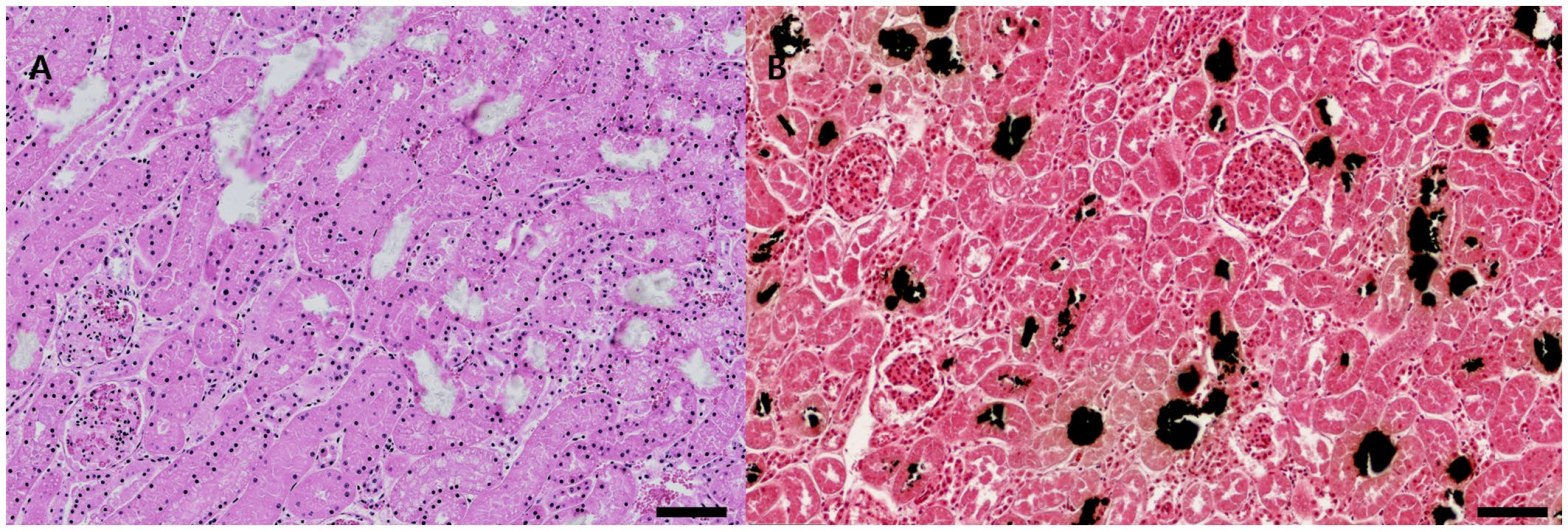
Renal lesions associated with ethylene glycol poisoning in a cat. **(A)** Kidney, cortex. There was diffuse acute tubular epithelial degeneration and necrosis with prominent tubular dilation and numerous, irregular, dark brown to black crystalline aggregates within tubular lumina, consistent with calcium oxalate crystals (hematoxylin and eosin stain, ×400; scale bar = 100 μm). **(B)** Kidney, cortex. Black stain representing calcium oxalate deposition (Yasue stain, ×400; scale bar = 100 μm).

The kidneys displayed acute tubular epithelial degeneration and necrosis with sloughing of tubular epithelium and prominent intraluminal deposition of crystalline material consistent with calcium oxalate. Crystals were most abundant in cortical tubules and collecting ducts and were accompanied by variable tubular dilation and mild interstitial congestion and inflammation (Figures 1A,B). No significant histologic lesions were observed in other organs.

In the Yasue staining method, calcified foci in renal tissue appeared as distinct black deposits against the surrounding parenchyma.

### Virological testing

PCR assays showed that pooled tissue samples (brain, spleen, lung, heart, kidneys, liver, and intestine) were negative for FIV, FeLV, FHV, and FCV, whereas FPV DNA was detected in the pooled tissue sample. Feline coronavirus RNA was detected in feces, indicating concurrent FCoV infection. No gross or histopathologic lesions characteristic of acute parvoviral enteritis or panleukopenia were observed.

### Toxicological findings

In the gastric tissue sample, ethylene glycol, glycolic acid and the internal standard GHB-D6 each formed their corresponding TMS derivatives and were clearly separated and detected by GC–MS.

**Figure 2:**
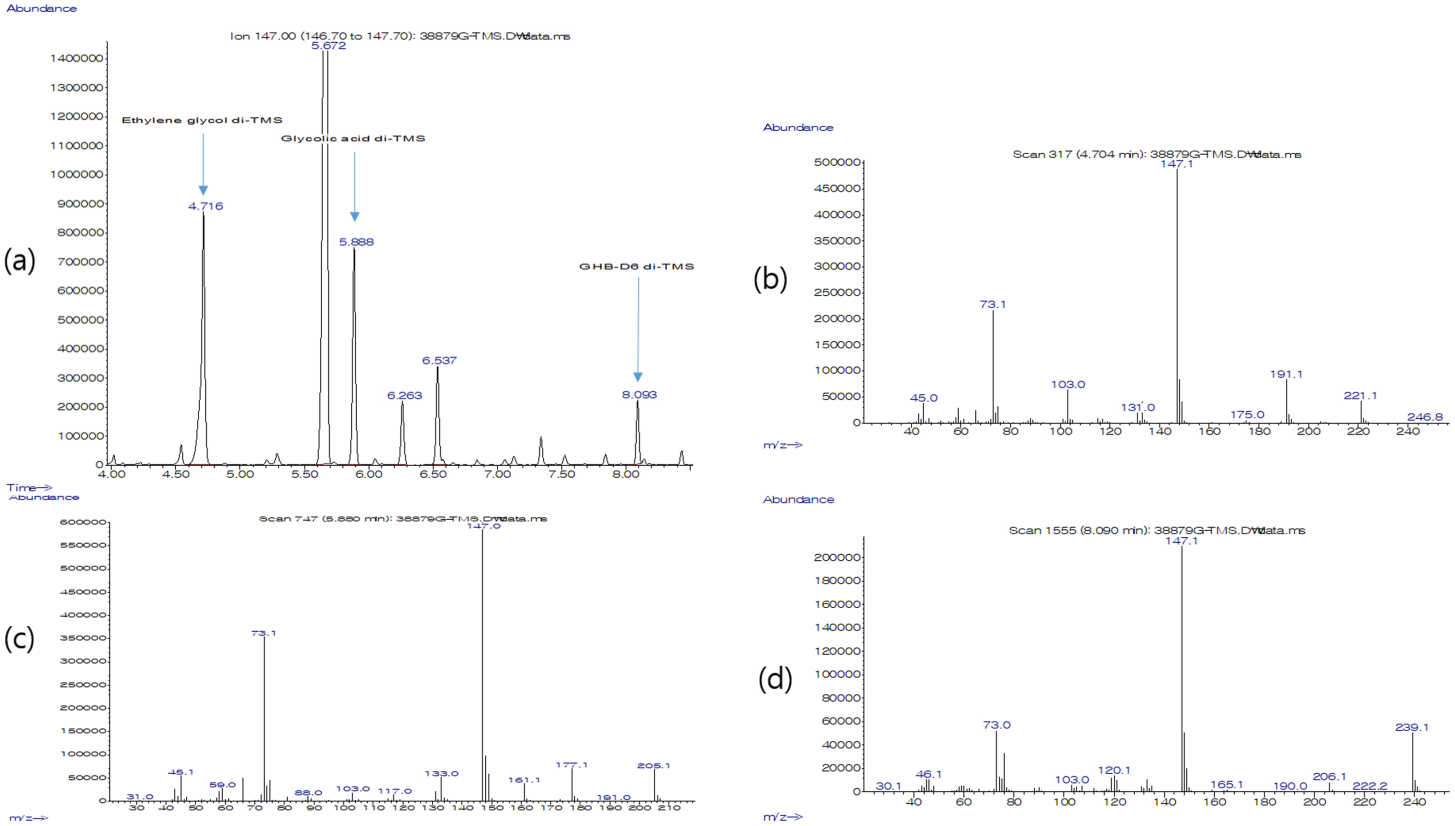
GC–MS analysis of gastric tissue from a cat with ethylene glycol poisoning. **(a)** Extracted ion chromatogram (*m/z* 147) obtained from derivatized gastric tissue, showing distinct peaks corresponding to ethylene glycol di-TMS (retention time 4.72 min), glycolic acid di-TMS (5.89 min), and the internal standard GHB-d6 di-TMS (8.09 min). **(b)** Electron impact mass spectrum of the ethylene glycol di-TMS peak at 4.7 min, characterized by a base ion at *m/z* 147 and fragment ions at *m/z* 45, 73, 103, 131, 191, 221, and 247, consistent with reference spectra. **(c)** Electron impact mass spectrum of the glycolic acid di-TMS peak at 5.9 min, with a base ion at *m/z* 147 and fragment ions at *m/z* 45, 73, 88, 103, 117, 133, 161, 177, and 205. **(d)** Electron impact mass spectrum of the internal standard GHB-d6 di-TMS peak at 8.1 min, showing a base ion at *m/z* 147 and characteristic fragment ions at *m/z* 46, 73, 103, 120, 165, 190, 206, 222, and 239. Together, these findings confirm the presence of ethylene glycol and its primary metabolite glycolic acid in the gastric sample and validate the analytical method used in this forensic investigation.

In the extracted ion chromatogram (m/z 147), ethylene glycol di-TMS eluted at 4.716 min, glycolic acid di-TMS at 5.888 min and GHB-D6 di-TMS at 8.093 min (Figure 2a). The electron-impact mass spectra of these peaks showed fragment patterns consistent with reference spectra for ethylene glycol di-TMS, glycolic acid di-TMS and GHB-D6 di-TMS (Figures 2b–d).

No other toxic agents, including cyanide, organophosphates, organochlorines, carbamates, rodenticides, barbiturates, benzodiazepines, phenothiazines, salicylates or alkaloids, were detected in the gastric sample. The GC–MS findings were interpreted qualitatively, and no matrix-based quantitative concentrations were generated for this specimen.

## Discussion

This case illustrates the critical role of veterinary forensic pathology and toxicology in determining the cause of death in suspected companion animal cruelty, particularly in intoxication scenarios where gross lesions may be subtle and clinical history is incomplete (1–3). Ethylene glycol poisoning is a well-recognized cause of fatal acute kidney injury in cats and dogs, but most published reports focus on clinical management rather than on its forensic implications within criminal investigations (5–9). By contrast, the present case was examined entirely postmortem at the request of law enforcement, and the diagnostic work-up was explicitly designed to generate court-admissible evidence on both the presence of a toxicant and its causal relationship to death (1–4).

From a pathologic standpoint, the findings in this cat were highly characteristic of ethylene glycol toxicosis. Although slightly overweight based on the body condition score, the animal was otherwise in good body condition. The main lesions consisted of bilaterally congested kidneys at necropsy and, histologically, acute tubular epithelial degeneration and necrosis with abundant intraluminal calcium oxalate crystal deposition within cortical tubules and collecting ducts. These lesions mirror established descriptions of ethylene glycol-induced nephrotoxicity, in which metabolism of ethylene glycol to glycolic acid, glyoxylic acid, and ultimately oxalic acid leads to severe metabolic acidosis and precipitation of calcium oxalate within renal tubules (5–8). The presence of these crystals strongly supports ethylene glycol poisoning when considered alongside the compatible history and analytical confirmation.

The toxicological findings provide the decisive link between the morphological lesions and a specific toxicant. Using a validated GC–MS method based on TMS derivatization, ethylene glycol and its primary metabolite glycolic acid were clearly detected in gastric tissue, along with the internal standard, consistent with previously reported analytical approaches. Because ethylene glycol is rapidly absorbed and distributed and has a relatively short half-life, its postmortem detection can be challenging, particularly when there is a delay between exposure and sampling or when only limited matrices are available (5–7). In this case, the choice of stomach tissue as the analytical matrix was strategic because it was likely to contain residual unmetabolized ethylene glycol relatively soon after ingestion and was available even when blood or urine were not. The simultaneous detection of ethylene glycol and glycolic acid, together with a chromatographic profile and mass spectra identical to reference standards, provides strong evidence of recent ingestion and supports a causal interpretation rather than mere environmental contamination. Because the present analysis was designed for forensic confirmation rather than tissue-level quantification, the findings should be interpreted as qualitative evidence of exposure and poisoning rather than as concentration-based toxicokinetic data.

An important forensic aspect of this case is the coexistence of viral nucleic acid detection in pooled tissue samples and feces. In free-roaming cats, exposure to and subclinical infection with parvoviruses and coronaviruses are common, and nucleic acids may be detectable even in the absence of overt disease. Had the investigation relied solely on virological testing, detection of FPV could have been overinterpreted as evidence of the primary cause of death. However, the lack of gross or histologic lesions typical of parvoviral enteritis or panleukopenia, coupled with the presence of severe ethylene glycol-type nephropathy and confirmed toxicant exposure, supports the interpretation that these infections were incidental and not the proximate cause of death. This highlights a recurring challenge in veterinary forensic work: differentiating between background findings and lesions directly responsible for death, especially in animals with multiple concurrent conditions (1–3).

Chain of custody and documentation are also central to the forensic value of this case. The carcass was formally submitted by the Seoul Gangbuk Police Station, with a specific case number and written request outlining the legal basis for examination under the national Animal Protection Act. All subsequent steps—reception, necropsy, sampling, laboratory analyses, and reporting—were recorded in an integrated case file. Such documentation aligns with recommended minimum standards for forensic investigations, which emphasize traceability of specimens, transparency of analytical procedures, and clear attribution of expert opinions to defined data sets (1–3). In legal proceedings, these elements can be as important as the scientific findings themselves, since deficiencies in chain of custody or reporting may undermine the evidentiary weight of toxicological results, regardless of their analytical robustness.

At a broader level, this case contributes to the emerging Korean literature on toxicological investigations in suspected companion animal cruelty. Recent retrospective studies from national and regional laboratories have documented a spectrum of toxicants identified in such cases, including anticoagulant rodenticides, pesticides, and various pharmaceuticals, and have emphasized the under-recognition of poisoning as a form of animal abuse (11–13). The present report extends this work by documenting a confirmed ethylene glycol poisoning with detailed pathological and analytical correlation and by demonstrating the feasibility of applying advanced GC–MS methods to routine forensic casework (6, 10, 11). Incorporating standardized toxicological protocols for community cats and other free-roaming animals found dead under suspicious circumstances could improve detection of similar cases and inform both preventive measures and prosecutorial decisions.

Finally, the methodological approach used here illustrates how research and casework can reinforce one another. The GC–MS procedure applied in this case was derived from a method originally developed for emergency human toxicology and has recently been adapted and validated for animal matrices, including feline serum and tissues. Applying such rigorously validated methods in forensic veterinary settings enhances analytical reliability and facilitates comparison of results across laboratories and species. Future multicenter collaborations that pool data from confirmed poisoning cases, including quantitative concentrations in different tissues and associated histologic findings, would allow more refined interpretation of toxicant levels in relation to survival times and clinical signs and would further strengthen the scientific foundation of veterinary forensic toxicology (1–3, 6, 10, 11).

## Conclusion

In a free-roaming cat submitted as part of an animal cruelty investigation, necropsy, histopathology and toxicological analysis converged to establish ethylene glycol poisoning as the cause of death.

This case illustrates how forensic veterinary pathology and toxicology can provide robust, court-ready evidence to support the investigation and potential prosecution of suspected animal poisoning offences.

## Data Availability

The raw data supporting the conclusions of this article will be made available by the authors, without undue reservation.
